# 

**Published:** 1905-04

**Authors:** 


### ATLANTA JOURNAL-RECORD OF MEDICINE

Successor to Atlanta Medical and Surgical Journal, Established 1855, and Southern Medical Record, Established 1870.

LIBRARY

SURGEON GENERAL'S OFFICE

JUL. 30-1906

192415

VOL. VII. ATLANTA, GA., APRIL, 1905. No. 1.

